# Mindfulness in surgeons

**DOI:** 10.1097/JS9.0000000000002909

**Published:** 2025-07-08

**Authors:** Valentina Bianchi, Carmen Nesci, Filomena Misuriello, Edoardo Piras, Anna Modica, Maria Michela Chiarello, Giuseppe Brisinda

**Affiliations:** a Division of Emergency and General Surgery, San Camillo-Forlanini Hospital, Rome, Italy; b Catholic School of Medicine “Agostino Gemelli”, Università Cattolica del Sacro Cuore, Facoltà di Medicina e Chirurgia, Rome, Italy; c Division of General Surgery, University of Trieste, Trieste, Italy; d Department of Surgery, Azienda Sanitaria Provinciale, Cosenza, Italy; e Division of Emergency Surgery and Trauma Center, Fondazione Policlinico Universitario Agostino Gemelli, IRCCS, Rome, Italy

Mindfulness is a discipline which is becoming increasingly common, nowadays^[1–4]^. This practice, which originated in the Eastern countries, especially where Buddhism was prevalent, was introduced in the Western scientific world, as Mindfulness-Based Stress Reduction (MBSR) program, in the late 70s^[5,6]^. It also evolved in Mindfulness-Based Cognitive Therapy (MBCT) more recently^[7]^. As stated^[5]^, it can be thought of as “moment-to-moment, non-judgmental awareness, cultivated by paying attention in a specific way, that is, in the present moment, and as non-reactively, as non-judgmentally, and as openheartedly as possible.” It is also considered a tool for facing sufferings of body and mind. In fact, many studies have been published on this topic and MBSR and MBCT roles on different pathological conditions, such as post-concussion symptoms^[8]^, chronic pain^[9]^, depression, and anxiety^[10]^.

A growing interest has arisen on its role on work stress and burnout for some categories^[11,12]^. MBSR can reduce stress, depression, and anxiety in psychiatric nurses^[13]^ and in nursing students^[14]^. It has some effects also on American law students^[15]^, military personnel^[16,17]^, and teachers^[18]^. But most of the studies are on healthcare professionals since they work in a high-stress setting. These studies aim to determine if mindfulness can improve workers’ resilience and reduce burnout and stress among these categories^[13,14,19–22]^.

Surgery is a high-stress reality, even more than other medical specialties^[23]^. This has also been measured using recordable body parameters, such as heart rates through a sensor patch during an operation^[24,25]^. The measurements also showed that surgeons acquire an adaptive response, since the variations are different between attending surgeons and residents. If a stress response during an operation, during which life and death decisions have to be made, is intuitive, there is also an environmental stress surgeons have to face. This is due to night shifts, long working hours^[26]^, short staffing, and medico-legal issues. A high percentage (about 40%) of American surgeons were burned out and 30% experienced symptoms of depression^[27]^. As well known, surgical complications have a deep impact on surgeons’ well-being, making the surgeon the “second victim” of an adverse event due to an operation^[28]^. In some specialties, surgeon well-being is even lower^[29]^. Considering these assumptions, we investigated the potential role of mindfulness and its effects on general surgeons and surgical residents. All the use of generative artificial intelligence in the article complies with the TITAN guideline^[30]^.

## What has been published in the literature?

A total of nine articles^[1,31–38]^ have been published. All of these articles, except two, were published by the authors from organizations based in the United States of America^[31–33,35,36]^ or by the authors from a UK organization^[34,38]^. Three of these studies were randomized controlled trials^[31–33]^, two no-randomized studies^[34,38]^, one a prospective cohort study^[1]^, and three pilot studies^[35–37]^. The target populations of the selected studies vary from surgical residents only (*n* = 7)^[1,32–35,37,38]^, surgeon (*n* = 1)^[36]^, or mixed (*n* = 1)^[31]^. Overall, the studies reported a positive impact of the implemented interventions (Table 1).

**Table 1:** Summary of the published studies

Author	Year	Number of participants	Intervention	Main results
Lebares *et al*^[33]^	2018	21	Feasibility of applying MBSR training in the daily clinical practice of surgical residents.	Feasibility and acceptance of mindfulness were demonstrated with long-lasting results over time.
Yusari *et al*^[37]^	2018	10	The students were randomly assigned to a control group or the MBSR group. The control group received no intervention at all. The MBSR group received mindfulness sessions for 15–20 min free of charge before the laparoscopic training.	The best improvement between the pre-tests and tests was achieved by the MBSR group for the color matching task, with an improvement of 72 ± 1.1 s. The MBSR group showed a better performance in all final tests compared with the control group (*P* < 0.05).
Lebares *et al*^[32]^	2019	21	Evaluation of modified-MBSR practices among first-year surgical residents.	The primary objectives were to evaluate the perceived stress and executive function. Other objectives were burnout, depression, motor skill performance, and changes in blood oxygen level-dependent functional neuroimaging. Modified MBSR in first-year surgical residents showed potential benefits for well-being and executive function.
Luton *et al*^[34]^	2021	14	ESRT in surgical trainees during the COVID-19 pandemic period.	Despite the pandemic period, ESRT proved to be feasible. Results showed a reduction in perceived stress and improved mindfulness at short-term follow-up in the intervention cohort.
Lebares *et al*^[31]^	2021	89	ESRT on perceived stress and cognitive function in first-year residents and specialist surgeons. Other outcomes included mindfulness and proinflammatory gene expression.	Participants treated with ESRT had a reduction of proinflammatory RNA expression compared to controls. ESRT may provide variable benefits, including stress reduction and improved cognition. Tailored courses could improve outcomes.
Saway *et al*^[36]^	2021	33	Feasibility and efficacy of MBSR.	Statistical analyses demonstrated an increase in awareness with a reduction in perceived stress. MBSR exercises are compatible with the OR practice and bring benefits both to surgeons and patients, reducing inattention during operation.
Burnet *et al*^[35]^	2023	85	Evaluate the prevalence of burnout among surgical residents and the effects of a weekly MBSR program.	Surgical residents suffer from high rates of burnout. Participating in the MBSR program was seen to reduce callousness, and residents appreciated it.
Luton *et al*^[38]^	2023	42	Influence of ESRT over a 2-year period in a single UK Statutory Education Body.	ESRT was associated with less stress and burnout, better mindfulness, and, most importantly, 13-fold better career progression.
Miazga *et al*^[1]^	2025	61	This was a prospective cohort study using a multiple methods design to assess a 12-week modified-MBSR curriculum tailored to busy surgical trainees involving a 30-min group session weekly and 15 min of home practice daily.	Twelve (20%) out of 61 eligible residents enrolled in the program and 8 (67%) completed the course. There was a statistically significant decrease in anxiety (*P* < 0.001) and an increase in surgical confidence (*P* = 0.007) following the mindfulness curriculum using validated survey tools. Thematic analysis identified that mindfulness tools were beneficial and regularly utilized by participants in the OR with sustained use 3 months post-intervention.

ESRT, Enhanced Stress and Resilience Training; MBSR, Mindfulness-Based Stress Reduction; OR, operating room.

However, due to the exiguity of the studies and the absence of large cohorts in most of them, definitive results and considerations cannot be made. Despite these limitations related to the execution of the studies and the number of participants, the feasibility of applying mindfulness in the clinical practice of surgical residents is documented. MBSR training did not have a negative effect on patient care and surgical training^[33]^. Similar results were found in a UK study evaluating the feasibility of mindfulness techniques in surgical trainees during the COVID-19 pandemic period^[34]^. The trainees in the cohort had a better career progression, a lower burn-out rate, and less stress^[38]^.

Burnet *et al* aimed to evaluate the prevalence of burnout among surgical residents and the effects of a weekly wellness program, including mindfulness practice^[35]^. A group of 85 residents was considered in the study. An optional 1-h conference per week was organized by senior surgeons, with time shielded from all clinical duties. Breakfast was also offered. The conference included a guided meditation and an open dialogue about the work experience, respecting the principles of tolerance, respect, anonymity, non-retaliation, compassion, and egalitarianism^[35]^. Participating in the program was seen to reduce callousness, and residents really appreciated a weekly conference on mindfulness^[35]^.

## The stress of the surgeon in the operating room

Operating room (OR) is a complex environment that requires the surgeon and all staff to succumb to unpredictable schedules and complex operations, often leading to the acquisition of bad habits. The stress experienced during an operation leads to distractions and errors in clinical practice. Several studies have shown that working on mindfulness improves attention. The administration of a 25-min lesson on the benefits of mindfulness and how to use a brief 4-min mindfulness practice before and after each operation resulted in a substantial increase in mindfulness and a reduction in perceived stress in healthcare and nursing staff in the OR.

Mindfulness exercises have been shown to be compatible with the activities of the OR and have brought benefits to both doctors and patients, reducing distraction during the operation^[36,39]^. More specifically, the mindfulness intervention was associated with a significant increase in mindfulness (*P* = 0.006) and flow state (*P* = 0.009) and a decrease in perceived stress (*P* = 0.03), particularly during the complex cases (*P* = 0.02). The results appear interesting, especially considering the fact that first-year residents were excluded from the evaluation because their lack of experience could have generated a bias.

On the other hand, a randomized trial evaluated the importance and effectiveness of mindfulness practices among 21 first-year surgical residents^[32]^. Modified MBSR seemed to increase well-being and executive function in residents. Despite the positive results documented by all studies, Miazga *et al* observe that for surgical residents, the greatest obstacle to participation in the mindfulness program was time^[1]^.

## Is a Zen surgeon possible?

Surgeons routinely operate under extreme stress and face the demands of high-stakes decision-making, long hours, and intensive performance standards^[40]^. In addition to having the knowledge and technical skills to practice surgery, an ideal surgeon needs to be alert, focused, and relaxed. He/she has to face the unexpected calmly. He/she is required to be flexible and resilient, unencumbered by previous cases or external situations, and warm and compassionate with patients, families, and colleagues.

Surgeons, like most humans, are typically not truly present. Our minds are commonly described as “monkey minds.” Humans tend to fantasize and ruminate about negative events, past failures, and future worries. The mind reacts to such events by resulting in anger, irritation, frustration, excitement, sadness, or disgust. This highly reactive and wild mind can cause depression, anxiety, addictions, and interpersonal conflicts. In short, the untrained mind is prone to being unhappy^[41,42]^.

Stephen and Mehta define mindfulness as the ability to focus attention on the present, accept external stimuli, and respond appropriately to them, even if they are outside our control^[43]^. They showed it reduces and protects against burnout and medical errors. Moreover, it increases professionalism. Four cornerstones of mindfulness are defined in the study: regularity of meditation, ability to appreciate and be grateful for the efforts made in the OR, regular mini meditations during the day, and management of negative thoughts. Including a mindfulness education plan in the surgeon’s clinical practice is fundamental in order to implement their academic productivity, performance in the OR and also for improving relationships with colleagues^[43]^. Overall, the selected studies demonstrated that engagement in mindfulness programs was beneficial for both surgical residents and surgeons. It is documented that mindfulness-based interventions are feasible in surgical contexts.

A study from New Zealand aimed to understand why mindfulness is important in a surgeon’s daily life^[44]^. They defined the ideal surgeon who is meant to be relaxed, resilient, and not affected by past failures. He should be attentive to what happens in the present, managing all unexpected events calmly. This is a prejudice. Reality is quite different. The surgeon often has to face negative events, but his/her mind can react by creating anxiety, depression, addictions, and interpersonal conflicts because of the nature of the human mind, which is unwilling to accept unhappiness. Mindfulness practices showed they can mitigate mood, subjective and objective health (improving hypertension and the immune system, reducing stress, anxiety, and depression). Medical errors are reduced, too. Constructive interactions with patients are promoted. Many surgeons practice mindfulness in the OR, naturally. Anyhow, its benefits can be extended to other aspects of daily life. The hectic life of the surgeon does not make mindfulness practices impossible. In fact, exercises are available online or in books and last a few minutes. And, they can give great benefits, even if administered in small doses continuously^[44]^. Furthermore, mindfulness seemed to reduce burnout rates, decrease medical errors, improve sleep, and even enhance surgical performance^[45]^.

No training school provides mindfulness courses, unfortunately. Anyhow, its introduction could be beneficial because of the stress surgeons are subjected to. So, the importance of mindfulness for improving surgeon performance, mindset, and interactions is reiterated in this study. Mindfulness is a practice that is becoming more and more popular among the public and which has demonstrated certain benefits in many working realities. In fact, according to many studies, mindfulness practice can decrease the stress levels and the burnout and depression rates. Its effect on healthcare workers has been debated, and its role is quite recognized^[46]^. We aimed to analyze the diffusion of mindfulness among general surgeons who work in a high-stress setting.

Despite the demanding assignment surgeons have, their practice appears to be feasible, especially for informal practice. In fact, a short session practice (i.e., 4–5 min) can be held even before an operation. Even traditional formal training (eight lessons lasting 90–120 min) seemed to obtain a certain adherence among surgeons.

Results on the reduction of burnout among these category members are a bit controversial, although promising. Other studies on a bigger cohort should be conducted to obtain definitive results. For sure, mindfulness could improve surgeons’ well-being, and their practice should be promoted during the specialty training and the daily practice (both for trainees and attending surgeons). Various studies promote protocols with different durations of practice and lessons (from 20 min to 2 h). Considering the clinical and surgical activities, shorter formal sessions and lessons (i.e., 20–30 min) could be easier to attend and could obtain better compliance. Experts in the field should be involved to define if an *ad hoc* protocol could be feasible and effective. A lesson about mindfulness or formal meditation could replace, every now and then, more well-accepted activities such as journal club or mortality and morbidity meetings, considering the positive effects they also have on surgical performance. Regular mini meditations must be encouraged and shown to be well accepted by surgeons.

Mindfulness can have a fundamental role on the well-being in the OR, but also other solutions must be thought of. Occupational health can offer access to counseling and support in some realities. This is helpful especially for surgeons who are sometimes the “second victim” of surgical complications^[28]^, have sleep deprivation and a poor work/life balance, and, sometimes, work in an environment where being brutalized by the public is not unusual and even bullying and undermining are not uncommon. In fact, according to this, 60% of the included surgical trainees reported they had this experience personally or as a witness in the workplace^[47,48]^. Proper job planning and mentoring can improve surgeon conditions.

## Conclusions

We found several studies supporting the relationship between mindfulness programs and better care provided by surgical residents and surgeons. Overall, the studies reported a positive impact of the implemented interventions. Despite the positive results documented by all studies, it was observed that for surgical residents, a barrier to participation in the mindfulness programs was time. Other affords have then to be made and mindfulness practice encouraged to create a surgeon who is not necessary “Zen” – considering mindfulness as a strategy to be relaxed or “Zen” instead of hungry or anxious is a mistake because it is more a way to recognize these emotions and how we react to them – but at least happy, satisfied, and unburned.

## Data Availability

The authors confirm that the data supporting the findings of this study are available within the article. All authors have given full approval of the version to be published.
